# Intermittent administration of a leucine-deprived diet is able to intervene in type 2 diabetes in *db/db* mice

**DOI:** 10.1016/j.heliyon.2018.e00830

**Published:** 2018-09-27

**Authors:** Siying Wei, Jingyu Zhao, Shuo Wang, Meiqin Huang, Yining Wang, Yan Chen

**Affiliations:** CAS Key Laboratory of Nutrition, Metabolism and Food Safety, Shanghai Institute of Nutrition and Health, Shanghai Institutes for Biological Sciences, University of Chinese Academy of Sciences, Chinese Academy of Sciences, Shanghai, 200031, China

**Keywords:** Physiology, Biochemistry, Microbiology

## Abstract

Continuous deficiency of leucine, a member of branched chain amino acids, is able to reduce obesity and improve insulin sensitivity in mice. Intermittent fasting has been shown to be effective in intervention of metabolic disorders including diabetes. However, it is unknown whether intermittent leucine deprivation can intervene in type 2 diabetes progression. We administered leucine-deprived food every other day in *db/db* mice, a type 2 diabetes model, for a total of eight weeks to investigate the interventional effect of intermittent leucine deprivation. Intermittent leucine deprivation significantly reduces hyperglycemia in *db/db* mice independent of body weight change, together with improvement in glucose tolerance and insulin sensitivity. The total area of pancreatic islets and β cell number are increased by intermittent leucine deprivation, accompanied by elevated proliferation of β cells. The expression level of Ngn3, a β cell progenitor marker, is also increased by leucine-deleted diet. However, leucine deficiency engenders an increase in fat mass and a decrease in lean mass. Lipid accumulation in the liver is elevated and liver function is compromised by leucine deprivation. In addition, leucine deficiency alters the composition of gut microbiota. Leucine deprivation increases the genera of *Bacteroides*, *Alloprevotella*, *Rikenellaceae* while reduces *Lachnospiraceae* and these changes are correlated with fasting blood glucose levels of the mice. Collectively, our data demonstrated that intermittent leucine deprivation can intervene in the progression of type 2 diabetes in *db/db* mice. However, leucine deficiency reduces lean mass and aggravates hepatic steatosis in the mouse.

## 1 Introduction

Leucine, one of the essential amino acids, belong to the family of branched-chain amino acids (BCAAs). There have been numerous interventional studies that have indicated that increasing dietary BCAAs including leucine has a health-beneficial effect related to obesity and type 2 diabetes (T2D) [1, 2, 3, 4]. However, paradoxically, increasing blood level of BCAA were recently found to be associated with increasing risks of T2D and insulin resistance [5, 6]. The blood BCAAs and their metabolites have been found to be promising biomarkers for metabolic disorders [7]. Two theories have been postulated to explain the potential detrimental effect of increasing blood BCAAs on glucose homeostasis [5, 6]. One is that elevation of BCAA level would stimulate mammalian target of rapamycin complex 1 (mTORC1), leading to uncoupling of insulin signaling via phosphorylation of insulin receptor substrate (IRS). The second hypothesis is that the mitotoxic metabolites of BCAAs, but not the BCAAs per se, causes mitochondrial dysfunction of β cells and aggravates T2D. Although it is still an unresolved issue regarding the molecular mechanism underlying the observed association of BCAA increase with T2D, current studies favor the second theory [6, 8].

Newgard et al. carefully investigated the effects of BCAAs using rats fed with high fat diet (HFD) for 15 weeks [9]. Supplemental BCAAs plus HFD caused insulin resistance, while paired feeding with HFD to match the calorie intake would not lead to insulin resistance [9]. It was also found that BCAA supplementation plus HFD led to chronic phosphorylation of mTOR and INS1 in the skeletal muscle and liver, suggesting a suppression of insulin signaling in these tissues. Consistently, it was found that restriction of BCAAs in Zucker-fatty rats is able improve insulin sensitivity in skeletal muscles via enhancing fatty acid oxidation and acyl-glycine export [10]. Deletion of BCATm, an enzyme that catalyzes the first step of BCAA metabolism, leads to increase in energy expenditure and improvement of insulin tolerance in the mice, in association with the activation of a futile protein turnover cycle [8]. Lately, it was found that the kinase and phosphatase that regulates branched-chain ketoacid dehydrogenase (BCKDH), a crucial enzyme for BCAA catabolism, can modulate glucose homeostasis in Zucker-fatty rats via ATP-citrate lyase [11].

A plethora of studies have indicated that continued leucine deprivation for a short time such as one week has a beneficial effect to improve insulin sensitivity. GCN2, an eIF2alpha kinase that can sense amino deficiency, is involved in the regulation of lipogenic pathway and fatty acid synthase (FAS) expression in the liver and consequently modulates fatty liver formation in response to leucine deprivation [12]. It was found that peripheral tissues are involved in the regulation of increased energy expenditure by acute leucine deprivation. Leucine deprivation increases lipolysis and decreases lipogenesis in white adipose tissue, while increases expression of uncoupling protein (UCP)-1 in brown adipose tissue BAT, suggesting increased thermogenesis [13, 14]. Acute leucine deprivation can increase hepatic insulin sensitivity via GCN2/mTOR/S6K1 and AMPK pathways as well as STAT5 [15, 16, 17]. Central nervous system is also involved in the modulation of energy metabolism by acute leucine deprivation. Corticotrophin-releasing hormone in the hypothalamus is implicated in the activation of sympathetic nervous system to enhance energy expenditure in response to acute leucine deprivation [18]. In addition, S6K1 and leptin signaling in the brain also regulate energy expenditure upon acute leucine deprivation [19, 20].

Although BCAAs and especially leucine play important roles in regulating glucose homeostasis, it is currently unclear if reducing dietary BCAAs is effective as an interventional strategy in improving insulin sensitivity and intervenes in T2D. Lately, intermittent fasting has been found be a promising way to improve β cell function and improve glycemic control in diabetic mice [21]. Intermittent fasting with a fasting-mimicking diet is able to intervene in the progression of diabetes in mice by increasing the number of β cells in the islets. Furthermore, intermittent fasting with a fasting-mimicking diet in humans is able to reduce the risk factors associated with metabolic disorders [22]. However, currently there is no study that combines the concept of “intermittent fasting” with “leucine deprivation”. In this study, we explored the idea whether or not intermittent leucine deficiency is able to intervene in the progression of T2D in a diabetic mouse model [6].

## 2 Materials and methods

### 2.1 Mouse model

Six-week-old male C57BL/ksJ-db (*db/db*) mice were purchased from SLAC (Shanghai, China) and maintained in single cage and in pathogen-free condition at the animal facility of Shanghai Institute for Biological Sciences (SIBS), Chinese Academy of Sciences (CAS). All mice were weighted at the beginning and randomly distributed to two groups: normal chow with free access to pair food and water (CTRL, n = 8) and intermittent leucine deprivation every other day (LEU-, n = 8). The pair food (with complete amino acids) and leucine-deficient food were obtained from Research Diets, Inc. (New Brunswick, NJ, USA; Cat A05080202 for L-amino acid rodent diet without added leucine and Cat A10021B for L-amino acid rodent diet). These diets were isocaloric and had the same composition in terms of carbohydrate and lipid. In addition, the cages were changed when diet was changed so that the mice had no access to their own fecal pellets. These experiments were conducted according to guidelines of the Institutional Animal Care and Use Committee of the Institute for Nutritional Sciences, SIBS, CAS with an approval number 2010-AN-8.

### 2.2 Blood glucose and insulin measurement

Mice were fasted for 6 h (9:00 a. m ∼ 15:00 p. m) before blood glucose measurements. Blood glucose was measured through tail vein using the OneTouch UltraEasy Blood Glucose Monitoring System (Lifescan, Milpitas, CA, USA). Serum insulin levels were measured by mouse enzyme-linked immunosorbent assay (Shanghai Enzyme-linked Biotechnology Co., Shanghai, China), according to manufacturer's instructions. Whole blood was withdrawn by removing eyeballs, and plasma was separated by centrifugation at 3,000 rpm for 15 min in EDTA-K2-treated microtubes (Kangjian Medical, Jiangsu, China). The homeostatic model assessment (HOMA) was the method used to quantify insulin resistance (HOMA-IR) and beta-cell function (%B). HOMA-IR was calculated using the following formula: HOMA-IR = (fasting glucose × fasting insulin)/22.5. HOMA %B was calculated using the following formula: HOMA-%B = (20 x fasting insulin)/(fasting glucose - 3.5)%.

### 2.3 Glucose tolerance testing (GTT) and insulin tolerance testing (ITT)

Before the test, mice were single-caged and fasted for 4 h for ITT (morning fasting) and fasted overnight for GTT. Glucose (2 g/kg) or insulin (2 unit/kg) was injected intraperitoneally. Blood glucose levels were measured at 0, 15, 30, 60, and 90 min after the injection.

### 2.4 Body composition analysis

Mice body composition was assessed at 2 month-old age by echoMRI (Houston, TX, USA) and the data of total fat mass and lean mass were recorded for each mouse, according to manufacturer's instructions.

### 2.5 Measurement of metabolic rate and physical activity

Mice at 2 month-of age were randomly allocated (n = 4 for each group) to testing of metabolic rate and physical activity by the comprehensive laboratory animal monitoring system (CLAMS-16, Columbus Instruments, OH, USA), according to manufacturer's instructions. Mice were allowed to adapt to the system for 24 h. Oxygen uptake (VO_2_), carbon dioxide production (VCO_2_), and respiratory exchange ratio (RER) were recorded in the following 24 h. Locomotion was monitored from the x-axis beam breaks.

### 2.6 Immunofluorescence analysis

Mice pancreas samples were collected and washed in PBS, then fixed in 4% paraformaldehyde overnight. After that, samples were dehydrated and embedded into paraffin. Finally, samples were sectioned into thick slices (4 μm). The sections were then deparaffinized in xylene and rehydrated through graded ethanol series (100%, 95%, 75% and PBS) followed by rinsing in distilled water. After heat-mediated antigen retrieval with 0.1 M citrate buffer (pH = 6.0), sections were blocked with blocking buffer (PBS + 1% normal goat serum + 0.1%trixton-100) for 1 h at room temperature. The following primary antibodies were used: anti-insulin (cat. no. C27C9, Cell Signaling Technology, Danvers, MA, USA), anti-glucagon (cat. no. ab10988, Abcam, Cambridge, UK), anti-Ngn3 (cat. no. sc-374442 from Santa Cruz Biotechnology, Dallas, Texas, USA), and anti-Ki67 (cat. no. 550609 from BD Biosciences, New Jersey USA). Sections were then incubated with primary antibodies in a humidified chamber overnight. After washing with PBS for 3 times, the sections were incubated for 2 h at room temperature with secondary antibodies (Alexa Fluor 488 donkey anti-rabbit IgG, or Alexa Fluor 546 donkey anti-mouse, dilution 1/500). All secondary fluorochrome-conjugated antibodies were purchased from Life Technologies (Thermo Fisher Scientific, Waltham, MA, USA). The nucleus was stained with Hoechst 33342 (Eugene Oregon, USA). The images were captured using a 40x objective with an LSM 510 confocal microscope (Zeiss, Jena, Germany).

### 2.7 Measurement of serum and hepatic parameters

Mice were euthanized *ad libitum* around 1p.m. on the control diet and the blood was immediately collected from the orbital sinus into EDTA-K2-treated microtubes (Kangjian Medical, Jiangsu, China). Then the microtubes were centrifuged at 3,000 rpm for 15 min and the supernatant serum was collected and were stored at −80 °C. Hepatic lipids were extracted with chloroform/methanol (2:1). Plasma levels of aspartate transaminase (AST) and alanine transaminase (ALT) were determined by AST/ALT Determination Kit (ShenSuo UNF, Shanghai, China). Plasma and hepatic levels of triglycerides (TG), total cholesterol (TC), were determined by corresponding kits (ShenSuo UNF, Shanghai, China). All of these assays were performed according to the manufacturer's instructions.

### 2.8 H&E staining of liver and pancreas samples

Mice liver and pancreas samples were collected and washed in PBS, then fixed in 4% paraformaldehyde overnight. After that, samples were dehydrated and embedded into paraffin. Finally, samples were sectioned into thick slices (4 μm). Then the samples were stained with hematoxylin and eosin (H&E). The images were captured using a 10x objective with an OLYMPUS BX51 microscope.

### 2.9 Antibodies and immunoblotting

The antibodies were purchased as follows: the antibodies against AKT, p-AKT, IR, p-IR, were from Cell Signaling Technology (Danvers, MA, USA). The antibodies against S6k and p-S6K were from Santa Cruz Biotechnology (Dallas, Texas, USA). The protocols for immunoblotting have been described previously (Feng et al., 2007).

### 2.10 Serum amino acids analysis

Mice serum was collected as previously described. Then serum samples were precipitated and diluted following by labeling with aTRAQ^TM^ Reagent Δ8. After that, we combined the aTRAQ^TM^ Reagent Δ8-Labled samples and aTRAQTM Internal Standard. Derivatized samples were introduced into Agilent 1200 LC system. AB SCIEX 4000 QTRAP LC-MS/MS system with TurbolonSpray ion source (Foster City, California, USA) were used for LC-MS/MS analysis. Internal standard was used to quantify the results.

### 2.11 Mice fecal samples collection

All mice were caged individually. Fresh fecal samples of mice were collected by tweezers in microtubes at 14:00–15:00 p.m. *at libitum* on a control diet to minimize possible circadian effects. Samples were collected into empty Eppendorf tubes on ice and immediately stored at −80 °C for future use.

### 2.12 Analysis of gut microbiota

Fecal DNA was extracted using the E.Z.N.A.® soil DNA Kit (Omega Bio-tek, Norcross, GA, U.S.) according to manufacturer's instructions. DNA concentration and purification were determined by NanoDrop 2000 UV-vis spectrophotometer (Thermo Scientific, Wilmington, USA), and the quality was checked using 1% agarose gel electrophoresis. The V3-V4 regions were then amplified. Then the PCR products were extracted from a 2% agarose gel and purified using the AxyPrep DNA Gel Extraction Kit (Axygen Biosciences, Union City, CA, USA). Purified products were pooled in equimolar and paired-end sequenced (2 ×300) on an Illumina MiSeq platform (Illumina, San Diego, USA) according to the protocols by Majorbio Bio-Pharm Technology Co. Ltd. (Shanghai, China). Raw fastq files were demultiplexed, quality-filtered by Trimmomatic and merged by FLASH. Operational taxonomic units (OTUs) were clustered with 97% similarity cutoff by using UPARSE (version 7.1 http://drive5.com/uparse/) and chimeric sequences were noted and deleted using UCHIME. The taxonomy of each 16S rRNA gene sequence was studied by RDP Classifier algorithm (http://rdp.cme.msu.edu/) against the Silva (SSU123) 16S rRNA database using confidence threshold of 70%.

### 2.13 Statistical analysis

All data are expressed as means ± SEM. Significant differences were assessed by two-tailed *Student'*s t test. Statistical tests were performed using Microsoft Excel (Microsoft, Redmond, WA, USA), R v3.3.2, or Prism6 (GraphPad Software, La Jolla, CA, USA) where appropriate.

## 3 Results

### 3.1 Intermittent leucine deprivation alters body composition, metabolic rate and physical activity in *db/db* mice

**Fig. 1:**
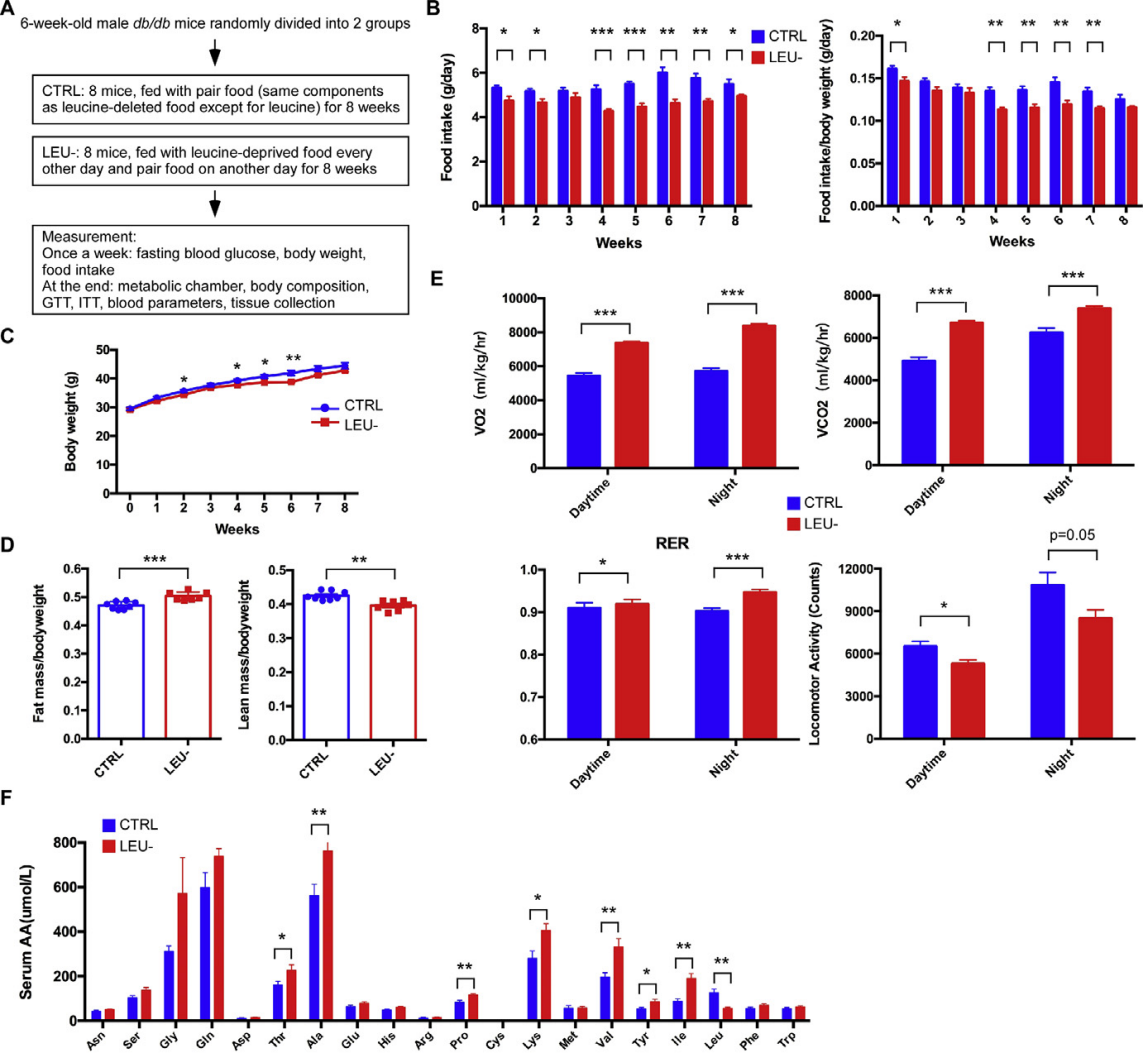
Intermittent leucine deprivation alters body composition, metabolic rate, physical activity, and blood amino acid levels in *db/db* mice. (A) Flowchart of the experiment. The *db/db* mice were divided into 2 groups. The control group (CTRL) was parallel-fed *ad libitum.* Another group was fed *ad libitum* with leucine-deprived food every other day (LEU-) groups (n = 8 mice/group). (B) Food intake of the mice. The adjusted food intake by body weight is shown on the right. (C) Body weight of the mice. (D) Quantification of fat mass and lean mass by MRI scan. (E) Analyses of the mice with metabolic chamber to quantitate O_2_ consumption, CO_2_ production, respiratory exchange ratio (RER), and X-locomotor activity. (F) Serum amino acid levels of the mice as detected by LC-MS/MS analysis. The mice were euthanized *ad libitum* on the control diet. Data are expressed as means ± SEM. *p < 0.05, **p < 0.01, ***p < 0.001.

Recently intermittent fasting has been postulated to be very effective in intervention of metabolic disorders [21, 23, 24, 25]. To investigate whether intermittent leucine deprivation also has a beneficial effect similar to intermittent fasting, we applied a strategy of leucine deprivation every other day to *db/db* mice that have been used as a classic model of type 2 diabetes (T2D) due to deficiency of leptin receptor. The *db/db* mice were divided into two groups in which one of them was fed with a diet deprived of leucine every other day; and the other group, serving as a control, was fed with a diet containing the same composition of nutrients except for the addition of leucine (Fig. 1A). Both groups were fed *ad libitum* without constraint of food intake. Overall, the food intake was slightly reduced in the leucine-deprived group as compared to the control group (Fig. 1B), as this is commonly observed in mice when eating leucine-deleted diet [26]. As a consequence, the body weight of leucine-deprived group was slightly decreased at certain time points during the 8-week-long experimental period, although the body weight at the end point of the experiment was not significantly different from each other (Fig. 1C). Analysis of body composition revealed that the leucine-deprived mice had a significant increase in fat mass and a significant decrease in lean mass (Fig. 1D), similar to the finding of a previous study [25]. Such observation is consistent with the notion that leucine is crucial for maintaining protein synthesis in the skeletal muscles [27]. We also analyzed the metabolic rate of the mice. The energy consumption of the mice measured by VO_2_ consumption and VCO_2_ production was significantly higher in the leucine-deprived mice than the control mice during both the daytime and night phases (Fig. 1E). Respiratory exchange ratio (RER) was significantly increased in the leucine-deprived mice compared to the control mice during both the daytime and night phases (Fig. 1E). In addition, physical activity was significantly decreased by intermittent leucine deprivation in the mice (Fig. 1E). Overall, these data indicate that intermittent leucine deprivation leads to complex metabolic changes in the *db/db* mice. The basal metabolic rate of the mice was increased by intermittent leucine deprivation. However, the physical activity was reduced by leucine deprivation, accompanied by reduction in lean mass.

In theory, leucine deprivation every other day would lower about 50% of leucine supply to the animal. We therefore measured the blood levels of amino acids. Mice were euthanized *ad libitum* around 1p.m. on a control diet. As expected, the blood leucine level was reduced to about 50% from 126.5 μmol/L to 57.2 μmol/L in average by intermittent leucine deprivation (Fig. 1F). Interestingly, the blood levels of other two BCAAs valine and isoleucine were significantly elevated by leucine deprivation (Fig. 1F). In addition, intermittent leucine deprivation significantly elevated the blood levels of a few other amino acids including threonine, alanine, proline, lysine, and tyrosine (Fig. 1F). These results, collectively, suggest the leucine deprivation is associated with increased levels of many other amino acids in the blood.

### 3.2 Leucine deprivation every other day reduces fasting blood glucose and improves insulin sensitivity

**Fig. 2:**
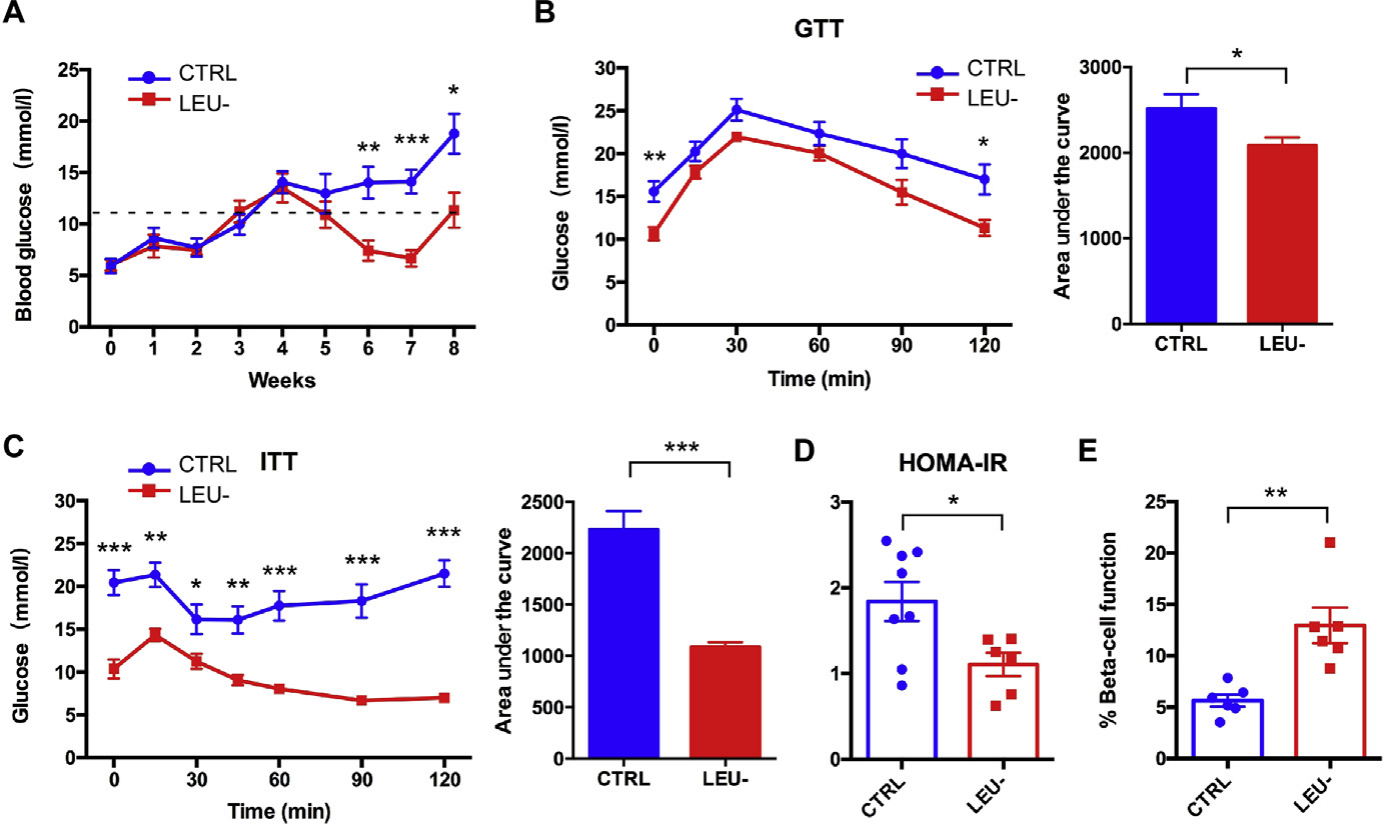
Intermittent leucine deprivation lowers fasting blood glucose level and improves insulin sensitivity in *db/db* mice. (A) Blood glucose levels of the two groups of mice (n = 8 mice/group). Blood samples were collected once every week. Mice were fasted for 6 h (morning fasting) before blood glucose measurement. A fasting blood glucose level of >11.1 mmol/L was defined as diabetic (dotted line). (B, C) Glucose tolerance test (GTT) and insulin tolerance test (ITT) at week 9. The area of under curve (AUC) is shown in the right panel for each test. (D, E) Homeostatic model assessment (HOMA) of insulin resistance (IR) and steady-state β-cell function (%B) at week 9. Data are expressed as means ± SEM. *p < 0.05, **p < 0.01, ***p < 0.001.

We next investigated whether or not intermittent leucine deprivation could impact glucose homeostasis. The *db/db* mice developed severe T2D over the experimental period shown as continuous increasing of fasting blood glucose level (Fig. 2A). However, intermittent leucine deprivation significantly prevented the development of T2D in these mice. Starting from the fifth week on, the fasting blood glucose level was significantly reduced by intermittent leucine deprivation (Fig. 2A). Consistently, glucose tolerance and insulin sensitivity of the mice as measured by GTT and ITT were both significantly improved by intermittent leucine deprivation (Fig. 2B and C). The calculated HOMA-IR was reduced by intermittent leucine deprivation (Fig. 2D). On the other hand, the calculated β-cell function was significantly elevated by leucine deficiency (Fig. 2E). These data, collectively, indicate that intermittent leucine deprivation is able to intervene in the development of T2D in the *db/db* mice.

### 3.3 The loss of β cells in the islets of *db/db* mice is prevented by intermittent leucine deprivation

**Fig. 3:**
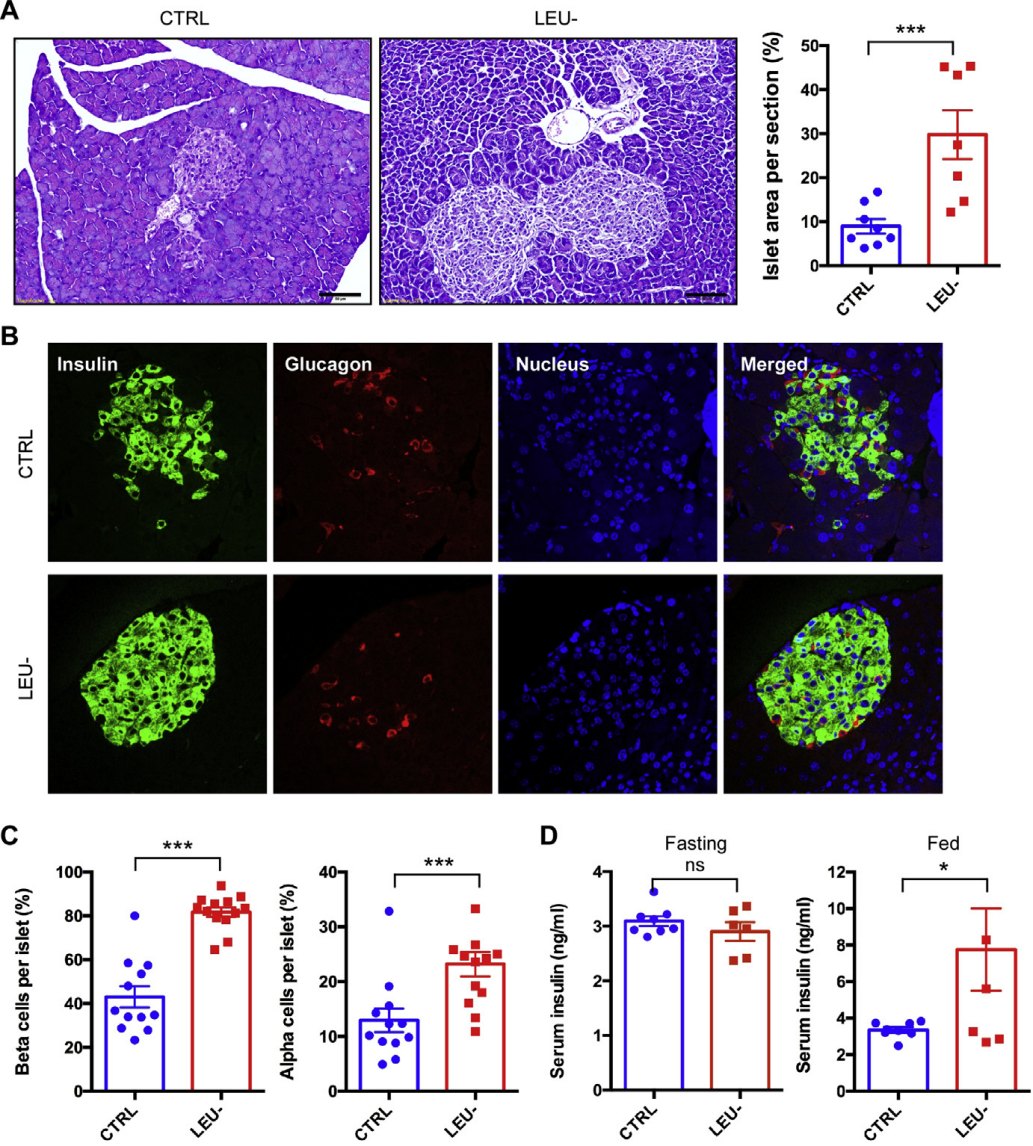
Pancreatic islet size and β cell number are improved by intermittent leucine deprivation. (A) Representative H&E staining of islets in pancreas sections. Scale bar: 50 μm. The ratio of islet area in the pancreas sections is shown in the right panel. (B) Representative immunofluorescence staining of pancreatic sections with antibodies against insulin and glucagon. The nucleus was stained with Hoechst 33342. Scale bar, 50 μm. (C) Quantitation of β cells and α cells per islet based on the immunofluorescence staining. (D) Serum insulin levels under both fasting and fed status. Blood samples were collected at week 9. Mice were fasted for 6 h for fasting condition (n = 8 mice/group). Data are expressed as means ± SEM. *p < 0.05, ***p < 0.001, ns for non-significant.

Development of T2D such as in *db/db* mice is accompanied by gradual loss of β cells in the pancreatic islets. We next investigated whether intermittent leucine deprivation affected pancreatic islets and β cells. H&E analysis with the pancreas sections revealed that the islet area was significantly elevated by intermittent leucine deprivation (Fig. 3A). Fluorescence staining of the pancreas sections with specific antibodies against insulin and glucagon demonstrated that the number of β cells were increased by intermittent leucine deprivation, together with an increase in the number of α cells (Fig. 3B and C). The blood insulin level under fasting condition was not significantly different between the two groups of the mice (Fig. 3D). However, the blood insulin level under fed condition was significantly increased by leucine deprivation (Fig. 3D). These data, therefore, clearly indicate that intermittent leucine deprivation is able to effectively prevent the loss of β cells in the *db/db* mice.

### 3.4 Intermittent leucine deprivation elevates β cell proliferation and Ngn3 expression in islets

**Fig. 4:**
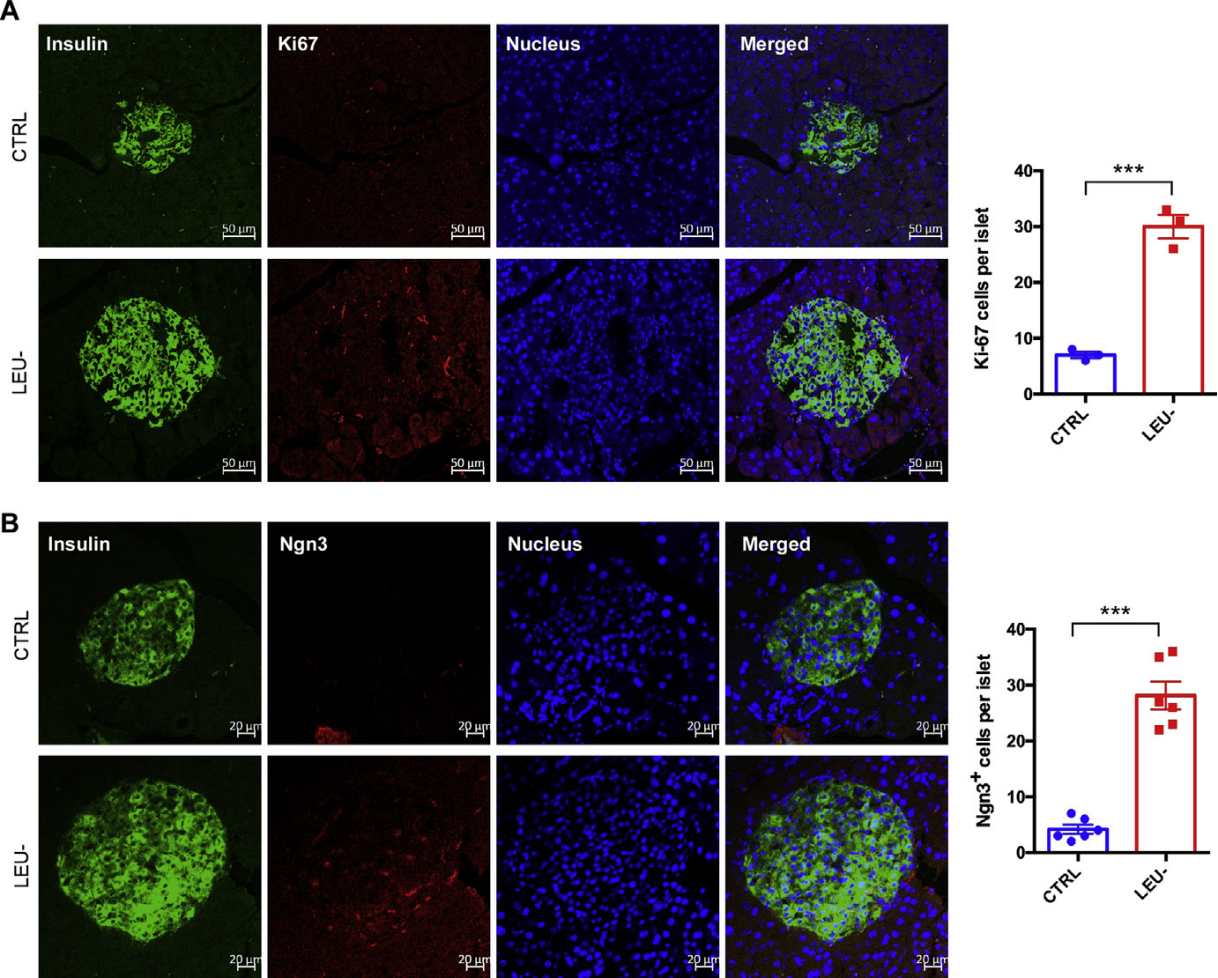
Intermittent leucine deprivation increases β cell proliferation and Ngn3 expression in islets. Representative immunofluorescence staining of pancreatic sections to detect Ki67 (A) and Ngn3 (B). Scale bar, 50 μm. The nucleus was stained with Hoechst 33342. Quantitation of the images is shown in the right panel. Data are expressed as means ± SEM. ***p < 0.001.

As we observed that the β cell damage in *db/db* mice can be prevented by intermittent leucine deprivation (Fig. 3), we next investigated whether the increase of the β cells in the mice is associated with changes of cell proliferation and Ngn3 expression, a marker for progenitors giving rise to β cells [21]. Immunofluorescent staining with Ki67 antibody, a marker for cell proliferation, revealed that the cell proliferation rate of the β cells was significantly increased by intermittent leucine deprivation (Fig. 4A). On the other hand, the staining of Ngn3 in the islets was also elevated by intermittent leucine deprivation (Fig. 4B). These data, therefore, indicate that intermittent leucine deprivation is able to increase β cell proliferation and elevate the number of β cell progenitors.

### 3.5 Hepatic steatosis and liver injury are aggravated by leucine deprivation

**Fig. 5:**
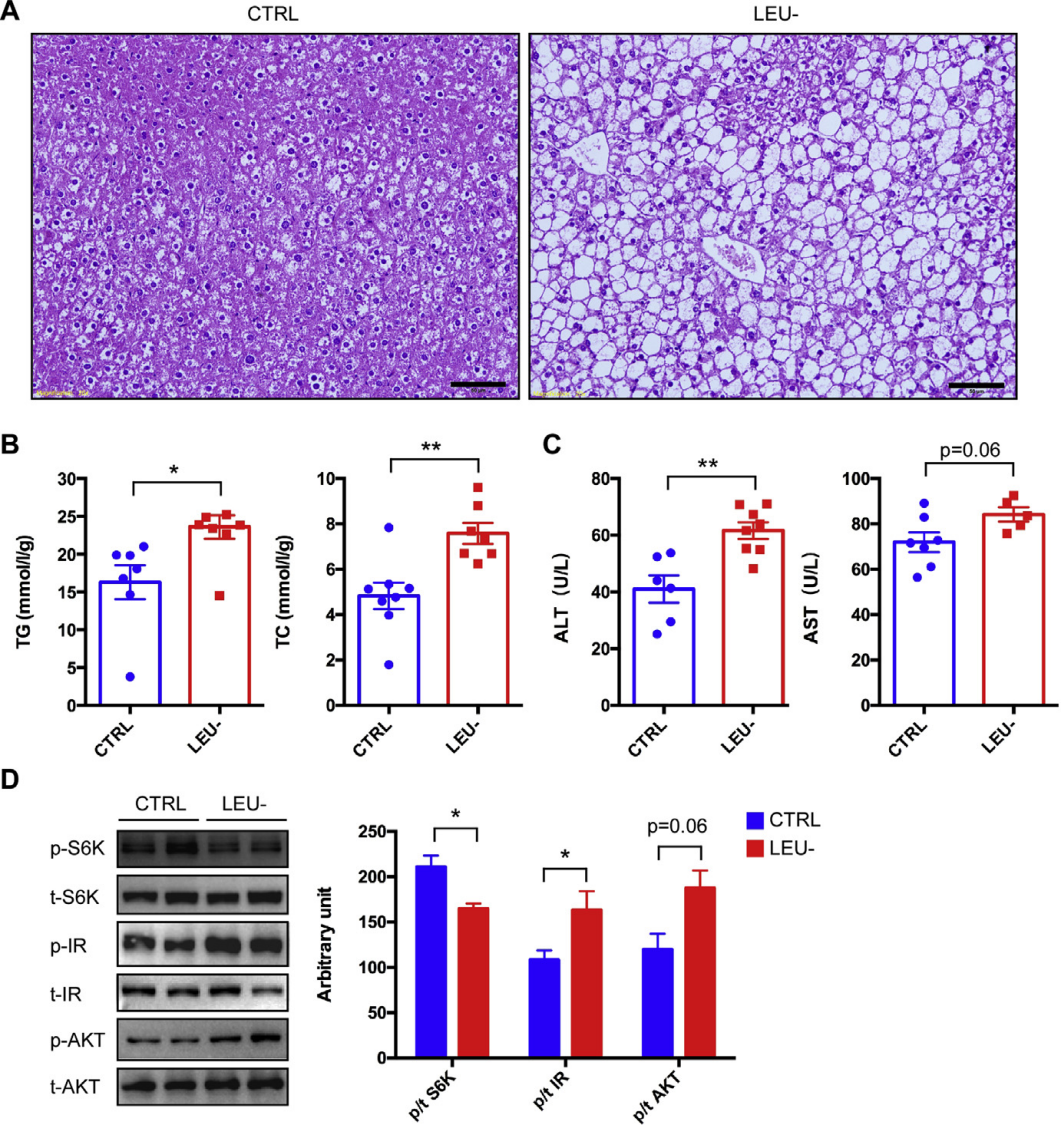
Intermittent leucine deprivation aggravates hepatic steatosis in *db/db* mice. (A) Representative H&E staining of liver sections. Scale bar: 50 μm. (B) Lipid levels in the liver. Triglyceride and total cholesterol levels were measured with the mouse liver tissues (n = 8 mice/group). (C) Analysis of liver function by measurement of blood levels of ALT and AST. (D) Western blotting analysis of liver samples. p stands for phosphorylated proteins and t for total proteins. Quantitation of the blots is shown in the right panel. Unaltered blots are shown in Supplementary Fig. 1. Data are expressed as means ± SEM. *p < 0.05, **p < 0.01.

Obesity is always associated with development of hepatic steatosis in animals. As *db/db* mice developed severe obesity over the course of the experiment, we analyzed whether intermittent leucine deprivation had an impact on liver functions. Surprisingly, H&E staining demonstrated that steatosis became much more obvious in the leucine-deprived mice than the control mice (Fig. 5A). Consistently, the liver triglyceride and total cholesterol levels were significantly elevated by leucine deficiency (Fig. 5B). In addition, the blood levels of ALT and AST were significantly increased by leucine deficiency (Fig. 5C). Collectively, these data indicate that liver steatosis and liver function are compromised by intermittent leucine deprivation in *db/db* mice.

We also analyzed the activities of mTORC1 and insulin signaling as both of them have been found to be modulated by leucine [15, 16, 17]. The phosphorylation of S6K, an indicator of mTORC1 activity, was downregulated by leucine deprivation in the liver tissue (Fig. 5D). In contrast, the phosphorylation of insulin receptor and AKT, indicators of insulin signaling, were both elevated by leucine deprivation in the liver tissue (Fig. 5D). These data, therefore, indicate that intermittent leucine deprivation results in a decrease in mTORC1 activity and an increase in insulin signaling in the liver, even though hepatic steatosis is aggravated by leucine deficiency.

### 3.6 Changes of gut microbiota by intermittent leucine deprivation

**Fig. 6:**
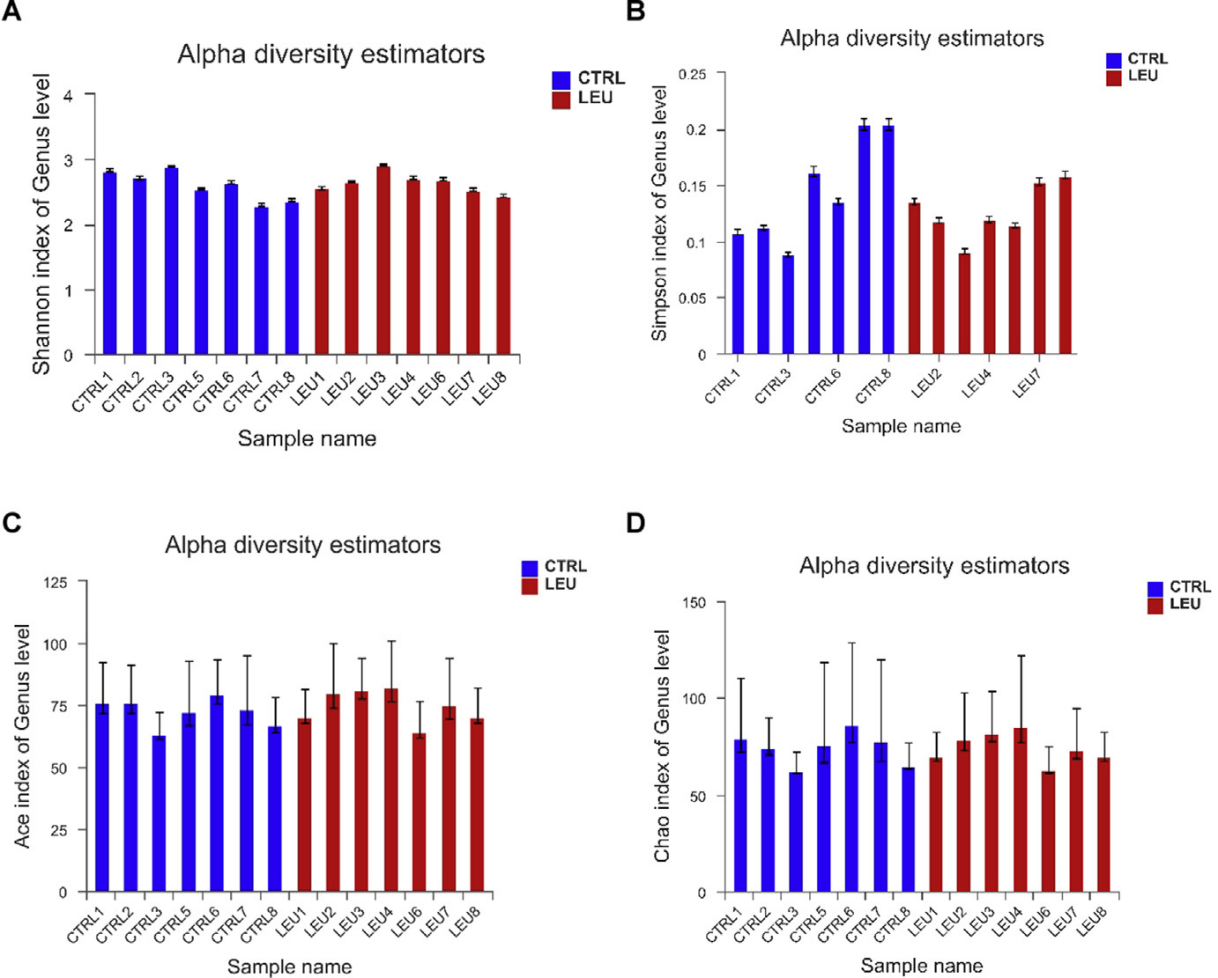
Alpha diversity of gut microbiota in the two groups of mice. Alpha diversity estimators of Shannnon index (A), Simpson index (B), Ace index (C) and Chao index (D) at genus level (n = 7 mice/group).

**Fig. 7:**
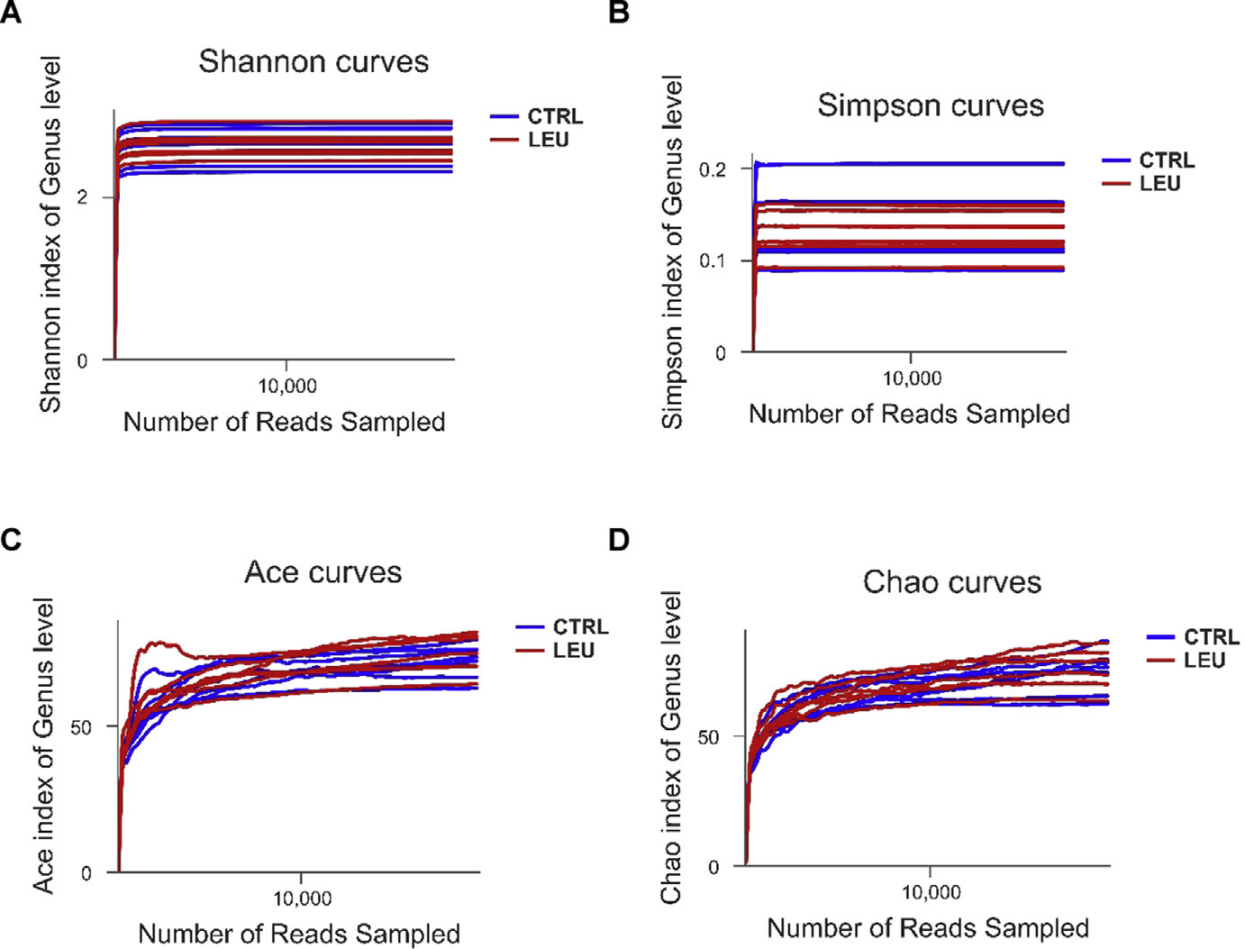
Rarefactions curve of gut microbiota in the two groups of mice. Rarefactions curves of Shannnon index (A), Simpson index (B), Ace index (C) and Chao index (D) at genus level (n = 7 mice/group).

In order to have a better understanding of the gut microbial profile of the mice, we then performed 16S rRNA gene analysis of mice fecal bacteria. Several indexes for alpha-diversity including Shannon, Simpson, Ace, and Chao indexes showed that there was no significant difference between the two groups (Fig. 6), indicating that the microbiome richness was not altered. As shown by respective rarefaction curves (Fig. 7), these curves became flatter to the right, meaning that the number of sequences analyzed was sufficient and the community of gut microbiota was fully represented.

**Fig. 8:**
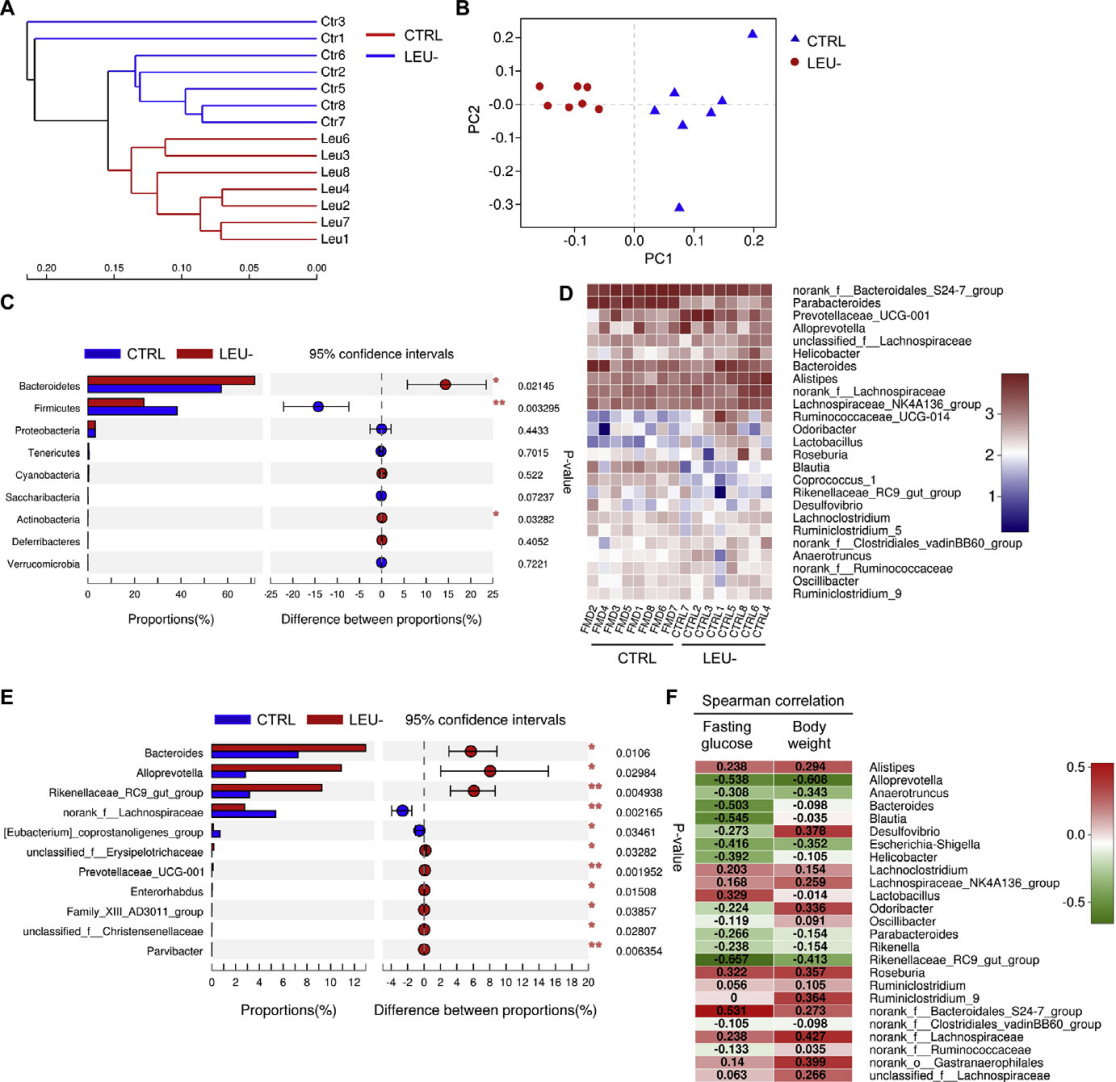
Intermittent leucine deprivation alters the composition of gut microbiota in db/db mice. (A) Hierarchical clustering of gut microbiota at genus level (n = 7 mice/group). (B) PCoA plot based on the relative abundance of microbiome at genus level. (C) Wilcoxon rank-sum test bar plot showing significant changes in the relative abundance at phylum level. The p value is shown in the right. *p < 0.05, **p < 0.01. (D) Heatmap showing relative abundance of bacteria at genus level. (E) Wilcoxon rank-sum test bar plot showing changes of bacteria at genus level. The p value is shown in the right. *p < 0.05, **p < 0.01. (F) Spearman correlation heatmap indicating the correlation of bacteria at genus level with fasting blood glucose and body weight. Correlation coefficient is shown inside.

**Fig. 9:**
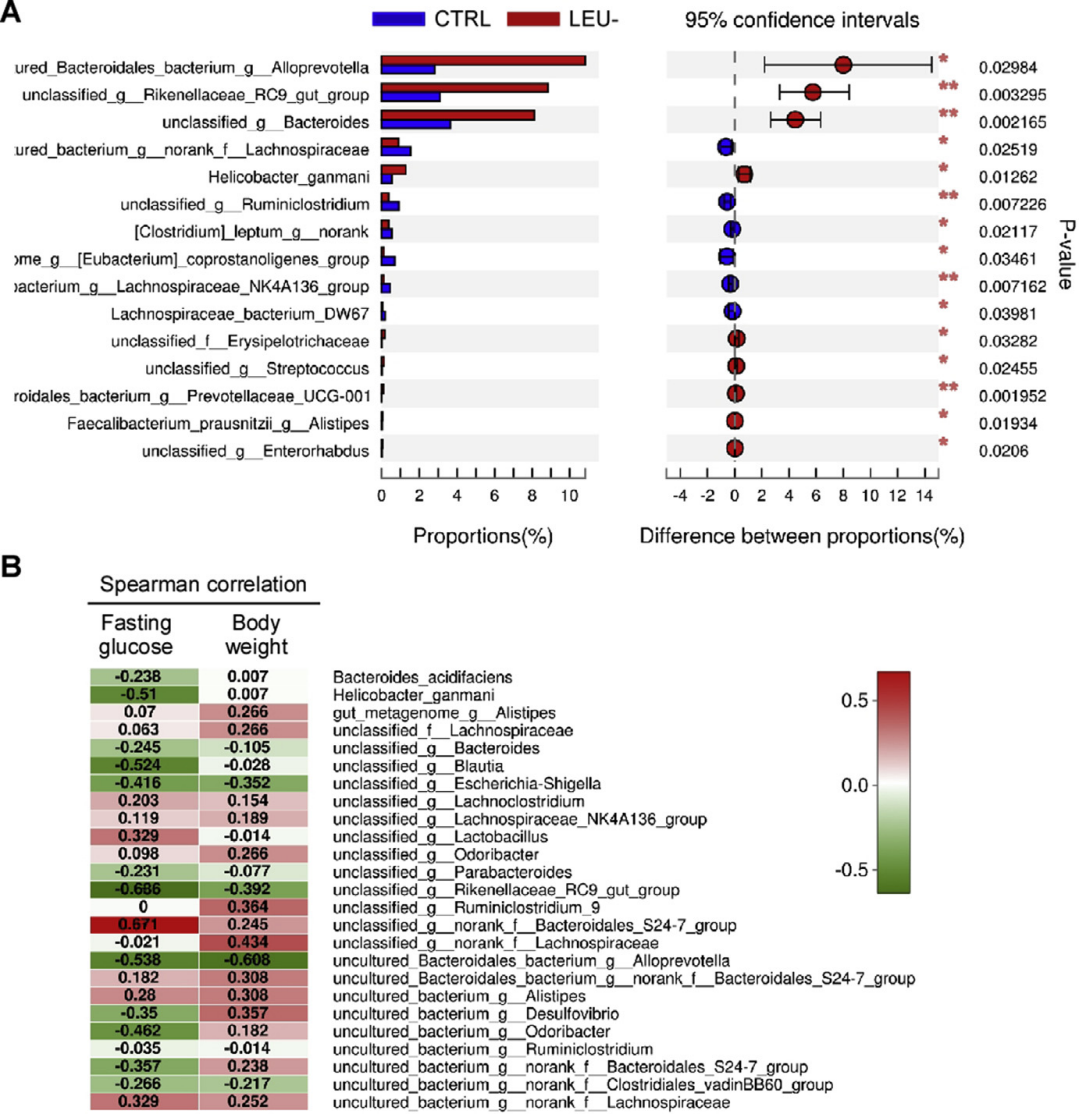
Intermittent leucine deprivation alters the composition of gut microbiota at species level. (A) Wilcoxon rank-sum test bar plot showing changes of bacteria at species level. The p value is shown in the right. *p < 0.05, **p < 0.01. (B) Spearman correlation heatmap indicating the correlation of bacteria at species level with fasting blood glucose and body weight. Correlation coefficient is shown inside.

The hierarchical clustering tree at genus level illustrated a structural rearrangement of gut microbiota after two months of intermittent leucine deprivation (Fig. 8A). Principal coordinate analysis (PCoA) of the relative abundance also confirmed a distinct compositional shift of the gut microbiome upon leucine deprivation (Fig. 8B). At phylum level, Wilcoxon rank-sum test revealed that intermittent leucine deficiency increased *Bacteroidetes* and *Actinobacteria* while reduced *Firmicutes* (Fig. 8C). Community heatmap analysis at genus level showed that alterations of numerous gut bacteria upon leucine deprivation (Fig. 8D). Detailed analysis using Wilcoxon rank-sum test revealed significant increase in the genera of *Bacteroides*, *Alloprevotella* and *Rikenellaceae RC9*, while *Lachnospiraceae* was significantly reduced (Fig. 8E). At species level, *Alloprevotella*, *Rikenellaceae RC9*, *Bacteroides*, and *Helicobacter ganmani* were significantly increased by leucine deprivation (Fig. 9A). In addition, *Lachnospiraceae* and *Ruminiclostridium* were significantly reduced by leucine deprivation (Fig. 9A). To further investigate whether these changes were correlated with the blood glucose level and body weight, we established a Spearman correlation heatmap at genus level with the data from individual mouse (Fig. 8E). *Bacteroides*, *Alloprevotella, Rikenellaceae RC9* and a few other genera were negatively correlated with fasting blood glucose level (Fig. 8F). *Alloprevotella* and *Rikenellaceae RC9* were also negatively correlated body weight (Fig. 8F). On the other hand, a few other genera such as *Alistipes* were negatively correlated with blood glucose level and body weight (Fig. 8F). Analysis at species level also showed that *Helicobacter ganmani*, *Blautia*, *Rikenellaceae RC9*, and *Alloprevotella* were negatively correlated with fasting blood glucose level, while *Bacteroidales S24-7* was positively correlated with the fasting blood glucose level (Fig. 9B). Together, these data indicated that intermittent leucine deprivation causes alterations of gut microbiota and some of these changes are correlated with the blood glucose level of the mice.

## 4 Discussion

Our study explored for the first time whether or not intermittent leucine deprivation can serve as an interventional strategy to intervene in T2D in the mice. We found that intermittent leucine deprivation is able to significant reduce hyperglycemia in *db/db* mice, accompanied by significant improvement in glucose tolerance and insulin sensitivity. The calculated HOMA-IR and β-cell function are also improved by intermittent leucine deprivation. The improvement of T2D in the mice appears to be a result of recovery of β cells in the islets. The total area of pancreatic islets is increased by intermittent leucine deprivation, together with significant elevation in β cells in the islets. The recovery of β cell function by leucine deficiency is also reflected by the increase of blood insulin level under fed state. We also investigated the potential mechanism underlying the recovery of β cells in the islets. Intermittent leucine deprivation can stimulate proliferation of β cells in the islets. In addition, the expression of Ngn3, a β cell progenitor marker, is also markedly increased by intermittent leucine deprivation. Collectively, our results have provided a proof-of-concept that intermittent leucine deprivation is potentially effective in intervene in T2D in the mice. It is noteworthy there that the improvement of T2D by intermittent leucine deprivation is not associated with reduction of obesity in the mice, as it was commonly accepted that reduction of obesity can improve insulin sensitivity. At the end of our experiment, intermittent leucine deprivation did not cause significant reduction of body weight. However, glucose homeostasis is significantly improved in the absence of body weight changes in response to intermittent leucine deprivation.

Numerous studies have pinpointed the importance of gut microbiota in type 2 diabetes [28, 29, 30]. However, whether or not leucine-deprived food can affect gut microbiota has not been characterized. 16S rRNA gene analysis of mice fecal bacteria revealed that the composition gut microbiome was altered by leucine deficiency. At phylum level, intermittent leucine deficiency increased *Bacteroidetes* while reduced *Firmicutes*. At genus level, *Bacteroides*, *Alloprevotella* and *Rikenellaceae RC9* was increased by leucine deprivation while *Lachnospiraceae* was reduced. Intriguingly, Spearman correlation analysis indicated that *Bacteroides*, *Alloprevotella, Rikenellaceae RC9,* and *Alloprevotella* were also correlated with the fasting blood glucose level. These findings are consistent with the observations reported by other group. Improvement of T2D in rats is associated with an increase in *Alloprevotella* that can produce short chain fatty acids with anti-inflammatory activity [31]. In humans, *Lachnospiraceae* was found to associate with T2D, while *Rikenellaceae* was linked to a healthier metabolic state in humans [32]. Intestinal colonization by a *Lachnospiraceae* bacterium could lead to development of T2D in obese mice [33]. The improvement of resveratrol on glucose homeostasis is associated with reduction of *Lachnospiraceae* in gut microbiome [34], very similar to our finding. Considering the potential contribution of gut microbiota to the development of diabetes, it will of great importance to explore how alterations of gut microbiome leucine underlie the interventional effect of deficiency improves T2D via alterations of gut microbiome in the future.

We also observed a number of “side effects” of intermittent leucine deprivation mainly in the skeletal muscle and liver. Leucine deprivation leads to an increase in fat mass, together with a decrease in lean mass. This observation is consistent with the idea that leucine is a crucial amino acid required to maintain synthesis of skeletal muscle proteins via stimulation of mTOR activity [27, 35]. As a matter of fact, numerous studies have indicated that supplementation of leucine is able to improve sarcopenia associated with aging [36, 37]. Analysis of the amino acids in the blood revealed that the blood leucine level is reduced as expected upon intermittent leucine deprivation. However, a number of other amino acids are significantly elevated upon leucine deprivation. This phenomenon is likely caused by an increase in muscle catabolism. The second major problem of intermittent leucine deprivation is aggravation of hepatic steatosis. Leucine deprivation is able to increase accumulation of lipids in the livers of the diabetic mice. In addition, the liver function is compromised by intermittent leucine deprivation. It is currently unknown why leucine deprivation is able to increase lipid accumulation in the liver. Previously it was found that short term leucine deprivation is able to reduce lipogenic pathway and fatty acid synthase (FAS) expression in the liver in a GCN2-dependent manner [12]. However, long-term leucine deficiency as shown in this study leads to aggravation of fatty liver in the mouse. It will be of importance to investigate in the future why the duration of leucine deficiency impacts differently on lipid metabolism in the liver.

To our knowledge, this is the first study aimed to explore the potential health benefit of long-term administration of intermittent leucine deprivation. Our results have both similarity and disparity with a few other studies to investigate long-term effect of a diet deficient in BCAA or leucine. Administration of a diet with all three BCAAs reduced by 45% in Zucker-fatty rats (ZFR) for 15 weeks reduced circulating levels of BCAA [10]. These ZFR fed with BCAA-deficient diet had no obvious improvement of glucose tolerance. However, the insulin sensitivity in the skeletal muscle was elevated, likely contributed by increases in fatty acid oxidation and acyl-glycine export [10]. Feeding of C57BL/6J mice with ∼2/3 reduction of all three BCAAs for 3 weeks are able to significantly improve glycemic control and insulin sensitivity, while reduces β cell metabolic stress [26]. Mice fed with a BCAA-deficient diet for 10 weeks gained less weight than the controls. Interestingly, the mice fed a diet deficient in ∼2/3 of leucine for 10 weeks ate less and had a trend of increased fat mass and decreased lean mass [26], similar to our observation in terms of body composition. However, whether or not deficiency of leucine alone affects glycemic control had not been investigated in that study [26]. Lately, it was found that ∼2/3 reduction of all three BCAAs led to rapid reduction in fat mass in mice fed with a high-fat, high-sugar diet, together with improvement of glycemic control [38]. It was also found that such a rapid loss of obesity in the obese mice is not caused by calorie restriction or increased activity, but by an increase in energy expenditure [38]. Likewise, we also observed that intermittent leucine deprivation is able to increase energy expenditure but in the meanwhile reduces physical activity. In another study, C57BL/6J mice were fed with a diet with 80% reduction of leucine for 8 weeks [39]. Leucine deficiency reduced body weight and fat mass percentage, while elevated lean mass percentage. Glucose tolerance was also improved by leucine deficiency in that study [39]. Collectively, most of the studies about long-term administration of leucine deficiency differ from our study in the way of diet administration. Our study is the first one that explored the idea of intermittent leucine deprivation. Different from other reports, the improvement of glycemic control using intermittent leucine deprivation is independent of reduction of obesity in our study. As it has been well accepted that improvement of insulin sensitivity always occurs after reduction of obesity, it can be argued that the observed beneficial metabolic effects upon leucine deficiency in most other studies is likely a result of body weight reduction. In other word, we postulate that the format of intermittent fasting including intermittent amino acid deprivation has an unique function to promote β cell function/regeneration, as supported by this study and a recent report by Longo's group [21]. In addition, as intermittent leucine deprivation is not associated with obesity reduction in our study, the fatty liver in the *db/db* mice is not improved but rather aggravated by leucine deficiency. In this respect, certain caution needs to be paid in the future to carefully optimize the dose and regime of leucine deprivation for diabetes intervention in order to minimize the potential “side effects”.

Interestingly, our study is consistent in certain degree with a recent report showing that short-term methionine deprivation was able to improve diet-induced obesity and T2D [40]. In contrast to our findings, methionine deprivation led to an increase in food intake and a decrease in fat mass [40]. In addition, hepatic steatosis was reduced by methionine deficiency [40]. These results are in agreement with findings that short-term leucine deprivation was capable of reducing fat mass and fatty liver [13, 14].

There are a number of limitations in our study that needs to be noticed for future exploration. First, this study was done with relatively young mice (6 weeks) before T2D was full-blown. It needs to be determined in the future whether or not intermittent leucine deprivation is able to effectively reverse T2D in older mice when they become diabetic. Second, we applied the intermittent leucine depravation for 8 weeks with the mice. It will be interesting to explore whether long term application of intermittent leucine depravation is still able to intervene in the progression of T2D. Third, it will be of importance to determine how long the beneficial effect of leucine deprivation would last when the leucine-deficient diet is no longer administered. Nevertheless, our study has provided a proof-of-concept that intermittent deprivation of an essential amino acid, instead of generalized intermittent fasting, is able to intervene in T2D in the mouse.

## Declarations

### Author contribution statement

Yan Chen: Conceived and designed the experiments; Analyzed and interpreted the data; Wrote the paper.

Siying Wei: Conceived and designed the experiments; Performed the experiments; Analyzed and interpreted the data; Wrote the paper.

Jingyu Zhao, Shuo Wang, Meiqin Huang, Yining Wang: Contributed reagents, materials, analysis tools or data.

### Funding statement

Yan Chan was supported by the National Natural Science Foundation of China (31630036 and 81390350), Ministry of Science and Technology of China (2016YFA0500103), Chinese Academy of Sciences (XDA12010102, QYZDJ495 SSW-SMC008, ZDRW-ZS-2016-8, Y817X11141, and CAS Interdisciplinary Innovation Team).

### Competing interest statement

The authors declare no conflict of interest.

### Additional information

No additional information is available for this paper.

## References

[1] Layman D.K., Walker D.A. (2006). Potential importance of leucine in treatment of obesity and the metabolic syndrome. J. Nutr..

[2] Zhang Y., Guo K., LeBlanc R.E., Loh D., Schwartz G.J., Yu Y.H. (2007). Increasing dietary leucine intake reduces diet-induced obesity and improves glucose and cholesterol metabolism in mice via multimechanisms. Diabetes.

[3] Guo K., Yu Y.H., Hou J., Zhang Y. (2010). Chronic leucine supplementation improves glycemic control in etiologically distinct mouse models of obesity and diabetes mellitus. Nutr. Metab..

[4] Li H., Xu M., Lee J., He C., Xie Z. (2012). Leucine supplementation increases SIRT1 expression and prevents mitochondrial dysfunction and metabolic disorders in high-fat diet-induced obese mice. Am. J. Physiol. Endocrinol. Metab..

[5] Newgard C.B. (2012). Interplay between lipids and branched-chain amino acids in development of insulin resistance. Cell Metab..

[6] Lynch C.J., Adams S.H. (2014). Branched-chain amino acids in metabolic signalling and insulin resistance. Nat. Rev. Endocrinol..

[7] Batch B.C., Shah S.H., Newgard C.B., Turer C.B., Haynes C., Bain J.R., Muehlbauer M., Patel M.J., Stevens R.D., Appel L.J., Newby L.K., Svetkey L.P. (2013). Branched chain amino acids are novel biomarkers for discrimination of metabolic wellness. Metabolism.

[8] She P., Reid T.M., Bronson S.K., Vary T.C., Hajnal A., Lynch C.J., Hutson S.M. (2007). Disruption of BCATm in mice leads to increased energy expenditure associated with the activation of a futile protein turnover cycle. Cell Metab..

[9] Newgard C.B., An J., Bain J.R., Muehlbauer M.J., Stevens R.D., Lien L.F., Haqq A.M., Shah S.H., Arlotto M., Slentz C.A., Rochon J., Gallup D., Ilkayeva O., Wenner B.R., Yancy W.S., Eisenson H., Musante G., Surwit R.S., Millington D.S., Butler M.D., Svetkey L.P. (2009). A branched-chain amino acid-related metabolic signature that differentiates obese and lean humans and contributes to insulin resistance. Cell Metab..

[10] White P.J., Lapworth A.L., An J., Wang L., McGarrah R.W., Stevens R.D., Ilkayeva O., George T., Muehlbauer M.J., Bain J.R., Trimmer J.K., Brosnan M.J., Rolph T.P., Newgard C.B. (2016). Branched-chain amino acid restriction in Zucker-fatty rats improves muscle insulin sensitivity by enhancing efficiency of fatty acid oxidation and acyl-glycine export. Mol. Metab..

[11] White P.J., McGarrah R.W., Grimsrud P.A., Tso S.C., Yang W.H., Haldeman J.M., Grenier-Larouche T., An J., Lapworth A.L., Astapova I., Hannou S.A., George T., Arlotto M., Olson L.B., Lai M., Zhang G.F., Ilkayeva O., Herman M.A., Wynn R.M., Chuang D.T., Newgard C.B. (2018). The BCKDH kinase and phosphatase integrate BCAA and lipid metabolism via regulation of ATP-citrate lyase. Cell Metab..

[12] Guo F., Cavener D.R. (2007). The GCN2 eIF2alpha kinase regulates fatty-acid homeostasis in the liver during deprivation of an essential amino acid. Cell Metab..

[13] Cheng Y., Meng Q., Wang C., Li H., Huang Z., Chen S., Xiao F., Guo F. (2010). Leucine deprivation decreases fat mass by stimulation of lipolysis in white adipose tissue and upregulation of uncoupling protein 1 (UCP1) in brown adipose tissue. Diabetes.

[14] Du Y., Meng Q., Zhang Q., Guo F. (2012). Isoleucine or valine deprivation stimulates fat loss via increasing energy expenditure and regulating lipid metabolism in WAT. Amino Acids.

[15] Xiao F., Yu J., Guo Y., Deng J., Li K., Du Y., Chen S., Zhu J., Sheng H., Guo F. (2014). Effects of individual branched-chain amino acids deprivation on insulin sensitivity and glucose metabolism in mice. Metabolism.

[16] Xiao F., Huang Z., Li H., Yu J., Wang C., Chen S., Meng Q., Cheng Y., Gao X., Li J., Liu Y., Guo F. (2011). Leucine deprivation increases hepatic insulin sensitivity via GCN2/mTOR/S6K1 and AMPK pathways. Diabetes.

[17] Yu J., Xiao F., Zhang Q., Liu B., Guo Y., Lv Z., Xia T., Chen S., Li K., Du Y., Guo F. (2013). PRLR regulates hepatic insulin sensitivity in mice via STAT5. Diabetes.

[18] Cheng Y., Zhang Q., Meng Q., Xia T., Huang Z., Wang C., Liu B., Chen S., Xiao F., Du Y., Guo F. (2011). Leucine deprivation stimulates fat loss via increasing CRH expression in the hypothalamus and activating the sympathetic nervous system. Mol. Endocrinol..

[19] Xia T., Cheng Y., Zhang Q., Xiao F., Liu B., Chen S., Guo F. (2012). S6K1 in the central nervous system regulates energy expenditure via MC4R/CRH pathways in response to deprivation of an essential amino acid. Diabetes.

[20] Zhang Q., Liu B., Cheng Y., Meng Q., Xia T., Jiang L., Chen S., Liu Y., Guo F. (2014). Leptin signaling is required for leucine deprivation-enhanced energy expenditure. J. Biol. Chem..

[21] Cheng C.W., Villani V., Buono R., Wei M., Kumar S., Yilmaz O.H., Cohen P., Sneddon J.B., Perin L., Longo V.D. (2017). Fasting-mimicking diet promotes Ngn3-driven beta-cell regeneration to reverse diabetes. Cell.

[22] Wei M., Brandhorst S., Shelehchi M., Mirzaei H., Cheng C.W., Budniak J., Groshen S., Mack W.J., Guen E., Di Biase S., Cohen P., Morgan T.E., Dorff T., Hong K., Michalsen A., Laviano A., Longo V.D. (2017). Fasting-mimicking diet and markers/risk factors for aging, diabetes, cancer, and cardiovascular disease. Sci. Transl. Med..

[23] Longo V.D., Mattson M.P. (2014). Fasting: molecular mechanisms and clinical applications. Cell Metab..

[24] Horne B.D., Muhlestein J.B., Anderson J.L. (2015). Health effects of intermittent fasting: hormesis or harm? A systematic review. Am. J. Clin. Nutr..

[25] Mattson M.P., Longo V.D., Harvie M. (2017). Impact of intermittent fasting on health and disease processes. Ageing Res. Rev..

[26] Fontana L., Cummings N.E., Arriola Apelo S.I., Neuman J.C., Kasza I., Schmidt B.A., Cava E., Spelta F., Tosti V., Syed F.A., Baar E.L., Veronese N., Cottrell S.E., Fenske R.J., Bertozzi B., Brar H.K., Pietka T., Bullock A.D., Figenshau R.S., Andriole G.L., Merrins M.J., Alexander C.M., Kimple M.E., Lamming D.W. (2016). Decreased consumption of branched-chain amino acids improves metabolic health. Cell Rep..

[27] Anthony J.C., Anthony T.G., Kimball S.R., Jefferson L.S. (2001). Signaling pathways involved in translational control of protein synthesis in skeletal muscle by leucine. J. Nutr..

[28] Qin J., Li Y., Cai Z., Li S., Zhu J., Zhang F., Liang S., Zhang W., Guan Y., Shen D., Peng Y., Zhang D., Jie Z., Wu W., Qin Y., Xue W., Li J., Han L., Lu D., Wu P., Dai Y., Sun X., Li Z., Tang A., Zhong S., Li X., Chen W., Xu R., Wang M., Feng Q., Gong M., Yu J., Zhang Y., Zhang M., Hansen T., Sanchez G., Raes J., Falony G., Okuda S., Almeida M., LeChatelier E., Renault P., Pons N., Batto J.M., Zhang Z., Chen H., Yang R., Zheng W., Li S., Yang H., Wang J., Ehrlich S.D., Nielsen R., Pedersen O., Kristiansen K., Wang J. (2012). A metagenome-wide association study of gut microbiota in type 2 diabetes. Nature.

[29] Karlsson F.H., Tremaroli V., Nookaew I., Bergstrom G., Behre C.J., Fagerberg B., Nielsen J., Backhed F. (2013). Gut metagenome in European women with normal, impaired and diabetic glucose control. Nature.

[30] Arora T., Backhed F. (2016). The gut microbiota and metabolic disease: current understanding and future perspectives. J. Intern. Med..

[31] Wei X., Tao J., Xiao S., Jiang S., Shang E., Zhu Z., Qian D., Duan J. (2018). Xiexin Tang improves the symptom of type 2 diabetic rats by modulation of the gut microbiota. Sci. Rep..

[32] Lippert K., Kedenko L., Antonielli L., Kedenko I., Gemeier C., Leitner M., Kautzky-Willer A., Paulweber B., Hackl E. (2017). Gut microbiota dysbiosis associated with glucose metabolism disorders and the metabolic syndrome in older adults. Benef. Microbes.

[33] Kameyama K., Itoh K. (2014). Intestinal colonization by a Lachnospiraceae bacterium contributes to the development of diabetes in obese mice. Microb. Environ..

[34] Sung M.M., Kim T.T., Denou E., Soltys C.M., Hamza S.M., Byrne N.J., Masson G., Park H., Wishart D.S., Madsen K.L., Schertzer J.D., Dyck J.R. (2017). Improved glucose homeostasis in obese mice treated with resveratrol is associated with alterations in the gut microbiome. Diabetes.

[35] Mitchell W.K., Wilkinson D.J., Phillips B.E., Lund J.N., Smith K., Atherton P.J. (2016). Human skeletal muscle protein metabolism responses to amino acid nutrition. Adv. Nutr..

[36] Leenders M., van Loon L.J. (2011). Leucine as a pharmaconutrient to prevent and treat sarcopenia and type 2 diabetes. Nutr. Rev..

[37] Woo J. (2018). Nutritional interventions in sarcopenia: where do we stand?. Curr. Opin. Clin. Nutr. Metab. Care.

[38] Cummings N.E., Williams E.M., Kasza I., Konon E.N., Schaid M.D., Schmidt B.A., Poudel C., Sherman D.S., Yu D., Arriola Apelo S.I., Cottrell S.E., Geiger G., Barnes M.E., Wisinski J.A., Fenske R.J., Matkowskyj K.A., Kimple M.E., Alexander C.M., Merrins M.J., Lamming D.W. (2018). Restoration of metabolic health by decreased consumption of branched-chain amino acids. J. Physiol..

[39] Lees E.K., Banks R., Cook C., Hill S., Morrice N., Grant L., Mody N., Delibegovic M. (2017). Direct comparison of methionine restriction with leucine restriction on the metabolic health of C57BL/6J mice. Sci. Rep..

[40] Yu D., Yang S.E., Miller B.R., Wisinski J.A., Sherman D.S., Brinkman J.A., Tomasiewicz J.L., Cummings N.E., Kimple M.E., Cryns V.L., Lamming D.W. (2018). Short-term methionine deprivation improves metabolic health via sexually dimorphic, mTORC1-independent mechanisms. FASEB J..

