# Supplementary figures and images for: Intermittent administration of a leucine-deprived diet is able to intervene in type 2 diabetes in db/db mice
Source: Heliyon. 2018 Sep 27;4(9):e00830. doi: 10.1016/j.heliyon.2018.e00830 (PMC6169254; doi:10.1016/j.heliyon.2018.e00830)

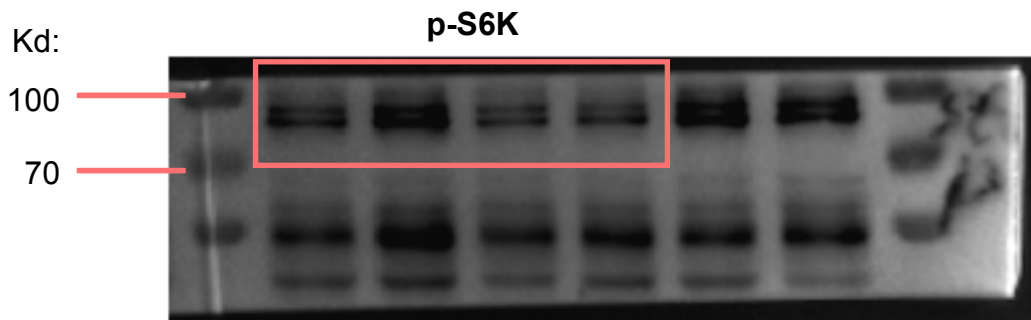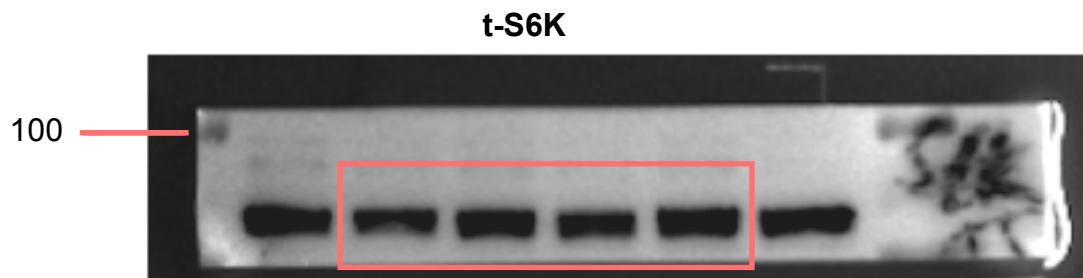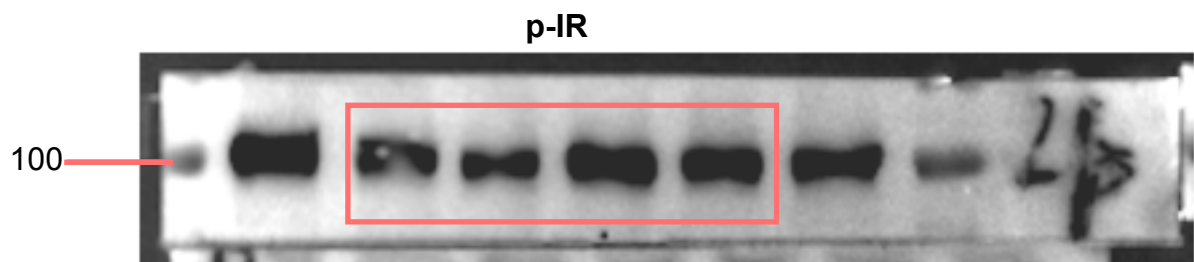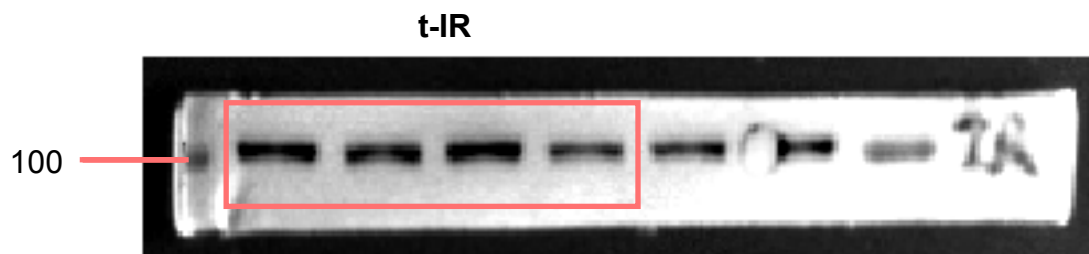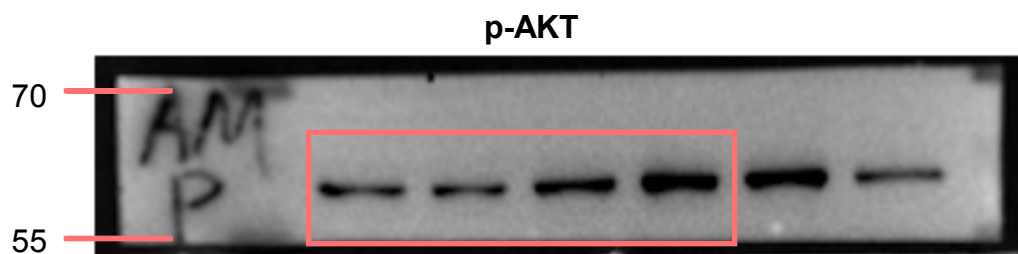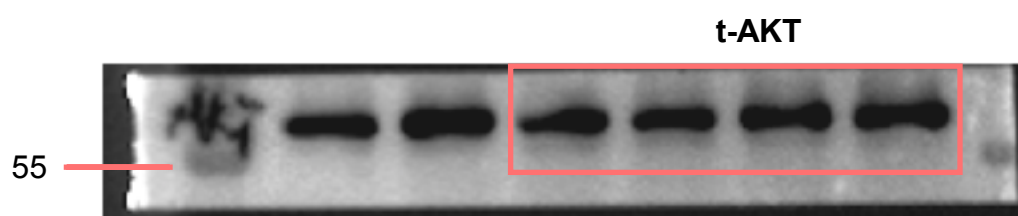

Supplement: Supplementary Figure 1 [file mmc1.pdf]
